# Redox Modulation in Therapy of Cancer: Some Pros and Cons

**DOI:** 10.3390/antiox14121496

**Published:** 2025-12-12

**Authors:** Ljubava D. Zorova, Dmitry S. Semenovich, Savva D. Zorov, Ilya P. Oleynikov, Anastasia S. Kargapoltceva, Dmitry V. Prutskikh, Polina A. Abramicheva, Irina B. Pevzner, Gennady T. Sukhikh, Dmitry B. Zorov

**Affiliations:** 1 A.N. Belozersky Research Institute of Physico-Chemical Biology, M.V. Lomonosov Moscow State University, 119991 Moscow, Russia; 7emenovich@gmail.com (D.S.S.); oleynikov.biophys@gmail.com (I.P.O.); abramicheva.polina@gmail.com (P.A.A.); pevzner_ib@belozersky.msu.ru (I.B.P.); 2 V.I. Kulakov National Medical Research Center of Obstetrics, Gynecology and Perinatology, 117997 Moscow, Russia; 3 Biological Faculty, M.V. Lomonosov Moscow State University, 119991 Moscow, Russia; kargapoltseva.nastya@mail.ru; 4 Research Institute of Molecular and Cellular Medicine, RUDN University, 117198 Moscow, Russia; steven.neb@mail.ru

**Keywords:** cancer, mitochondria, redox potential, glutathione, NADH, NAD, uncouplers, uncoupling, reticulum, fragmentation, membrane potential, sensor

## Abstract

Redox potential controls a vast array of biochemical reactions, and its changes influence the transition from normal to pathological states. However, cellular redox potential is primarily assessed after extraction of water-soluble components (reduced and oxidized) from biological material, particularly glutathione, which, due to its abundance, determines intracellular redox potential. This process involves mechanistic averaging of redox potential values across tissue or cell, although existing data suggest, and sometimes directly indicate, heterogeneity in redox potential both within cells and within tissue. We argue that mitochondria determine cellular redox state, in particular through changes in the state of the mitochondrial reticulum caused by various internal and external factors. We describe the possibilities for regulation of redox status of the cell and organ as a potential therapy for various pathologies, particularly cancer, and propose intensifying efforts to utilize *intrinsic* redox indicators. We specifically examine the possibility of changes the redox potential in cancer cells through the use of oxidative phosphorylation uncouplers and propose mechanisms by which cancer cells may be killed using uncouplers. Particular attention is paid to the mitochondrial membrane potential as a powerful regulator of cellular metabolism, possibly unrelated to the regulation of reactive oxygen species levels, with the possible existence of a membrane potential sensor in cells.

## 1. Introduction

The study of the dependence of biological system functioning on the degree of reduction/oxidation of its components is widespread due to the obvious relevance of this relationship to both the normal course of biochemical reactions and the occurrence of pathologies. While admitting the importance of the redox environment for the functioning of a complex biological system, it should be recognized that this is only one of the factors that determines the rate of a biochemical reaction in a cell. Given the vast number of high-quality studies on the influence of the redox environment on the course of reactions (e.g., see [1,2,3,4,5,6,7,8,9]), we will limit ourselves to a brief description of the fundamental principles and indicate alternative mechanisms for the influence of the biological system’s environment (be it a cell or subcellular structure) on the course of biochemical reactions. However, typical errors arise from incomplete perception of the spatial organization of cellular components and physico-chemical processes catalyzed or occurring spontaneously. In this analytical review, we trace the 50-year evolution of the RedOx discipline, starting from the seminal findings of the mid-1970s, characterized by the establishment of the energetic characteristics of mitochondria and the development of technical approaches to assess redox status. The current fundamental and practical status of redox science will be presented. Although the primary focus is on the relevance of this discipline to oncology, many details we describe particularly those related to mitochondrial functioning could be relevant to other biomedical problems.

## 2. What Elements Create and Maintain Redox Potential in the Cell?

**Figure 1:**
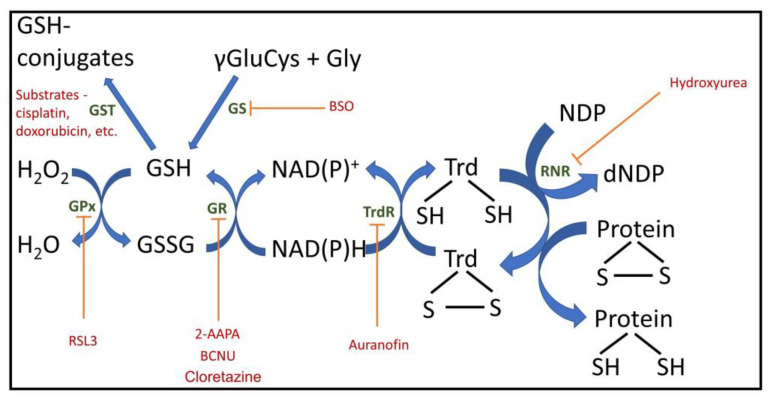
Important cellular redox components with inhibitors and regulators. GPx, glutathione peroxidase; GR, glutathione reductase; Trd, thioredoxin; TrdR, thioredoxin reductase; GS, glutathione synthesis; GST, glutathione S transferase; RNR, ribonucleotide reductase; NDP, nucleoside diphosphate; dNDP, deoxy nucleoside diphosphate; 2-AAPA, 2-acetylamino-3-[4-(2-acetylamino-2-carboxyethylsulfanylthiocarbonylamino)phenylthiocarbamoylsulfanyl]propionic acid, GR inhibitor; BCNU, carmustine, 1,3-bis(2-chloroethyl)-1-nitrosourea, GR inhibitor; BSO, buthionine sulfoximine, GS inhibitor; RSL3, (1S,3R)-3-(2-chloroacetamido)-4-oxo-1-(4-(trifluoromethoxy)phenyl)cyclohexane-1-carboxylic acid), GPx4 inhibitor.

One common problem is an incomplete understanding of the components that organize redox homeostasis. Glutathione, rightfully the primary focus of attention, is the main water-soluble substance that determines redox potential in the cell [10,11]. In a normal cell, almost 99% of the glutathione pool is in its reduced form (GSH) [10]. As a result, the entire redox potential of the cell is largely reduced and determined by glutathione. The prevalence of the reduced form of glutathione prevents components of the biological system (proteins, lipids, nucleic acids, and small molecules) from converting into an oxidized state during oxidative stress, a condition characterized by acute and powerful generation of oxidants, primarily reactive oxygen species (ROS). This condition threatens the development of pathologies associated with oxidative stress, and glutathione, both at its site of synthesis (the cytoplasm) and in secondary compartments (mitochondria, reticulum, nucleus, etc.) after its transfer from the cytosol to organelles and to the extracellular volume [12], serves as a kind of guarantee to prohibit unwanted oxidative changes. Therefore, reduced glutathione, reflecting redox environment plays an exceptional role in cellular metabolism, determining the main processes, including proliferation, death and differentiation, the disruption of which is fraught with the development of various pathologies, including cardiovascular, neurodegenerative, and immune, as well as cancer, and aging. In glutathione, the redox-sensitive component is the SH group of its cysteine residue. Due to its chemical properties, this SH group can undergo changes, in particular by binding to proteins through this group, resulting in the loss of its redox-regulating activity. Similar SH groups are also present in cellular proteins, both membrane-bound and, where possible, freely mobile within the aqueous phase of the cell or the exterior (e.g., in albumins or globulins). It should be noted that it is the pool of these free SH groups in proteins, due to their abundance, that determines the overall redox potential of the system. Thus, the intracellular or intra-organelle pool of redox-forming components consists of mobile and immobile redox buffering elements. Along with glutathione, the NAD(P)H/NAD(P) pair, their coupled substrates (e.g., lactate and pyruvate), as well as pairs of reduced/oxidized flavin coenzymes with their coupled substrates (e.g., succinate and fumarate), make an important contribution to the overall redox buffer in the cell. Redoxins, which act as redox sensors that significantly regulate cellular redox status, also make an important contribution. These redoxins, which belong to the same subfamily, include peroxiredoxins, thioredoxins, and glutaredoxins (Figure 1). Their activity leads to changes in the redox state of target proteins through the reversible oxidation of their thiol groups [13].

Thioredoxin reductase, which mediates NADPH-dependent oxidation of substrates, is crucial for establishing and managing the redox status of the system. Thioredoxins themselves are a family of proteins whose redox state largely regulates the state of the entire biological system. The reactions catalyzed by thioredoxins are very similar to those catalyzed by glutathione and include SH—S-S transitions in proteins and are involved in DNA synthesis (reviewed in [14]).

## 3. How Homogeneous or Heterogeneous Is the Redox Potential in a Cell?

**Figure 2:**
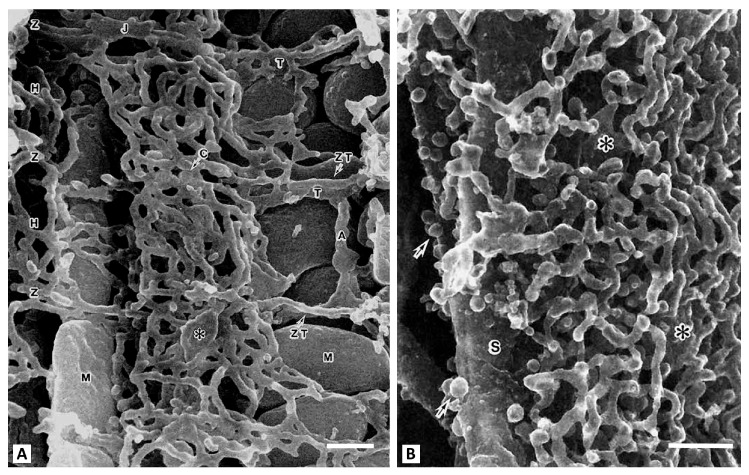
Scanning electron microscopy of the contents of a myocyte from a rat cardiac ventricle. (**A**), center of the cell. T-tubules (T) run transversely at the Z-band level and are connected to sacroplasmic reticulum (SR) network. The axial tubule (A) connects two T-tubules. Representatives of two mitochondrial populations (M) are visible. A vertical row of interfibrillar mitochondria is on the left, and on the right are subsarcolemmal mitochondria. Around the mitochondria (M), the SR forms simple meshes at the H-zone (H) and Z-disk (Z) levels. The asterisk shows large cisternae (C). (**B**), the space adjacent to the sarcolemma (S). This slightly oblique view shows the sarcotubules forming multilayered polygonal meshes. Large plate-like cisternal SR are intercalated among them (shown by asterisks). Arrows indicate numerous caveolae (from [19] with permission). Bar, 0.1 µm.

Today, it is extremely difficult to quantitatively assess the redox potential in a multicomponent biological system. When working with a single enzyme, the redox potential and subsequent assessment of its effect on functioning is evaluated by setting fixed values of the oxidation-reduction potential in the in vitro environment and varying the ratios of oxidized and reduced equivalents (for example, by adding a ferro/ferricyanide pair in different proportions) [15]. However, evidence suggests that even this scheme is complex. The difficulty of assessing the redox environment arises even for single enzymes, where the individual reactivity of their thiols can be observed even in an averaged over space redox environment. An example is glyceraldehyde phosphate dehydrogenase (GAPDH), whose redox-sensitive components (Cys152 and Cys156 [16,17]) exhibit different modification capacities. This suggests the possibility of different redox potentials in the environment of these relatively closely located amino acid residues. The presence of many similar facts for other enzymes suggests heterogeneity in the redox environment of individual enzymes. This significantly determines the regulation of enzymatic activity, depending on the degree of reduction of redox-regulated residues both in the active center and on the periphery of the enzyme, allosterically influencing enzymatic activity. It is quite easy to understand such an uneven distribution of redox components and redox buffers in the cell, if we take into account that the cellular contents, which include macromolecules organized into structures, are highly structured. In this situation, the diffusion-controlled processes are extremely limited due to the crowding of intracellular structures in different cells and in the extracellular space (as an example, see Figure 2). Even if we assume that the average water content in a cell to be about 2/3 of the total volume, the presence of very large hydrophobic and hydrophilic surfaces of intracellular structures with the ability to structure water molecules, leads to a limitation of diffusion-controlled processes in the cell [18]. For individual enzymes, water molecules can reach the enzyme’s active sites, creating a water space with limited water mobility. This can be a determining factor in enzymatic activity, regulating substrate availability and the removal of enzymatic products. It is relevant to dissolved in water solutes including mobile redox buffers (such as glutathione) thus limiting their flux and leading to a non-stationary redox state of mobile redox buffers during active metabolism. Figure 2B suggests that intracellular diffusion limitations for mobile redox buffers may begin just beneath the cell membrane, adjacent to the highly branched membrane network of the sarcoplasmic reticulum. It may restrict the movement of water-soluble redox buffer, possibly causing uneven distribution of redox potential from the cell membrane and deeper in the cell.

Recently, we proposed that extended mitochondria (mitochondrial reticulum) are a kind of immobilized redox buffer due to that the membrane potential on the inner mitochondrial membrane of the mitochondrial reticular system [20] is the same, and it is in equilibrium with the redox potential of the environment of this reticulum. The membrane potential can also be considered a buffer because it is in thermodynamic equilibrium with the cytosolic ATP levels reflecting a basic principle of ATP synthase functioning [21]. Inside the cell, the ATP content is very high (several millimoles), which is quite paradoxical, because these values are much higher than the affinity of ATP-utilizing enzymes for ATP. As a result, the catalytic centers of these enzymes are completely saturated with ATP, which calls into question the catalytic regulatory role of ATP. However, the millimolar concentrations of ATP in the cytosol creates a highly buffered energetic environment, and the membrane potential of the mitochondria acts as a regulatory factor. Together, these components (ATP and the membrane potential) ensure the maintenance of optimal homeostasis and are a determining element in the formation of a healthy cellular phenotype [22,23].

In healthy cell phenotype, mitochondria often form a single network [24,25,26], ensuring a rapid response to changes in the redox potential of the environment. In this case, the rate of response to such changes corresponds to the rate of membrane potential propagation along the mitochondrial network, in contrast to the slow distribution of intracellular mobile buffers such as glutathione. In the pathological cell phenotype, the mitochondrial reticulum is fragmented, and each fragment has its own membrane potential and corresponding redox environment [27]. We emphasize the particular importance of mitochondria in maintaining redox potential in the cell, with needs to be further assessed in the future.

This capability, in most cases, is generally absent when exploring a cell or organ. However, the cell has intrinsic redox indicators that can sometimes be used, although this is associated with methodological difficulties, primarily due to the fact that the optical spectra of these indicators overlap with the spectra of many other cellular components, and light scattering is an issue in such measurements and their interpretation.

**Figure 3:**
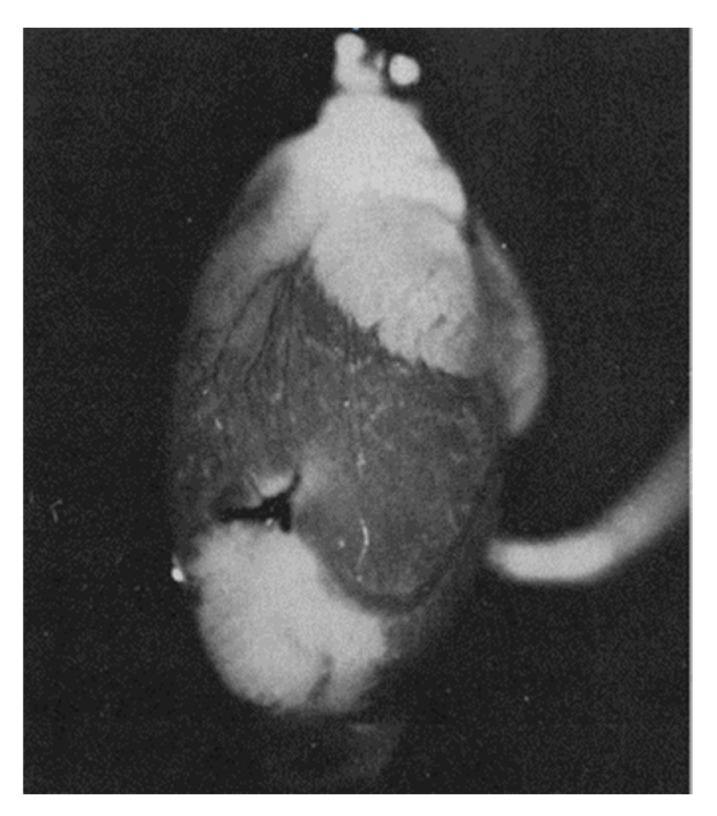
NADH fluorescence emission from a perfused rat heart with a local ischemic area near the apex (seen as white areas) caused by ligation of a coronary artery (white zones correspond to areas with increased NADH fluorescence, corresponding to ischemic zones in which respiration is suppressed due to lack of oxygen) (from [42] with permission).

Britton Chance suggested using a number of intrinsic indicators, in particular reflecting the oxygen content in the cell, such as myoglobin, cytochrome oxidase, flavins, and NAD(P). He was one of the founding fathers of bioenergetics, demonstrating that reduced NADH and reduced flavins are the main reducing equivalents supplying electrons to the mitochondrial respiratory chain, determining respiration and ATP synthesis [28]. The optical spectra of these components are sensitive to redox changes; therefore, if there is a precise and unambiguously interpretable system for measuring these changes, they can individually be good reporters of the redox state of their environment. Despite the many technical difficulties of assessing redox status in vivo, B. Chance’s work left an important mark in bioenergetics and redox biology. He developed equipment and approaches for the adequately assessment of NADH and oxidized flavoprotein (FP) fluorescence, including FAD, to study the metabolic state of mitochondria, and the work included clinical studies [29,30,31,32,33,34,35]. The basic points for such work were the proof that a high metabolism leads to a decrease in NADH with a parallel increase in the level of FP, ultimately leading to a lower NADH/FP ratio. It was B. Chance who introduced into practice an approach that allows using the fluorescence ratio of NADH and FP as a sensitive indicator of the redox state of mitochondria [30,31,32,33,34,35,36,37,38,39,40,41]. This is well demonstrated by Figure 3, which shows the result of one of these studies, in which an isolated heart was subjected to ischemia caused by cessation of perfusion [42]. It shows how ischemic zones in the heart can be visually distinguished by assessing NADH fluorescence.

**Figure 4:**
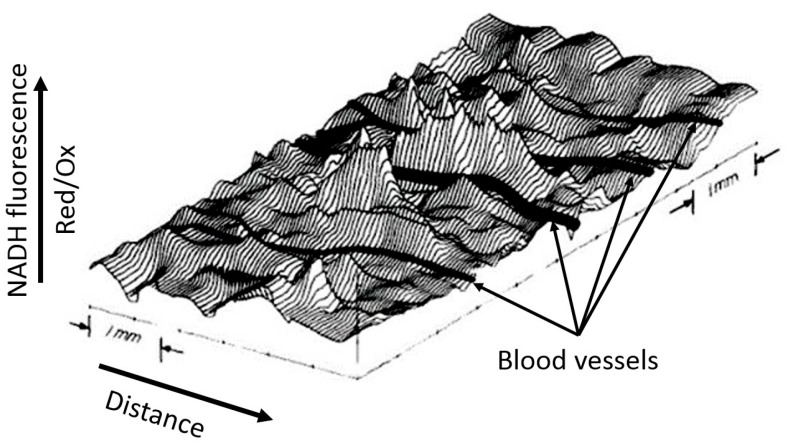
Spatial distribution of NADH fluorescence of the cortex area of an intact brain under normoxia. A computer plot of corrected NADH fluorescence over the exposed surface of the rat cerebral cortex in normoxia is given. The area measures approximately 6.0 mm × 3.5 mm. Blood vessels (in black) drawn in by hand run perpendicular to contour lines and are all veins draining into the sagittal sinus (from [43] with permission).

Although the example shown in Figure 3 is visually binary in nature without smooth transitions (NADH is either reduced or moderately oxidized) due to the limited ability of the equipment to quantitatively evaluate the fluorescence diversity, the steady-state levels of reduction in respiratory carriers can vary significantly depending on the environment, as we have argued above. We deliberately emphasize the dependence of the redox potential assessment on the environment, since ignoring the possible heterogeneity of the redox potential throughout the cell and tissue is a classic mistake made by researchers, creating great difficulty in the overall assessment of the redox state of the system. In another study conducted by B. Chance’s group (Johnson Research Foundation, University of Pennsylvania), the use spectral characteristics of the surface of an open normal brain showed significant heterogeneity in the degree of NAD reduction all over the entire studied area (Figure 4). Such heterogeneity clearly characterizes the unequal metabolic (respiratory) activity of different parts of the organ (in this case, the brain), only partially depending on the proximity to blood vessels.

## 4. How Effective Is Cancer Therapy with Redox Agents?

There is an opinion, not fully supported by experimental evidence due to the insufficient assessment of the diversity of cancer phenotypes that the cytoplasmic redox state of cancer cells is more oxidized than that of homologous, but non-tumor, cells. This opinion undermines that tumor cells have higher ROS levels than non-tumor cells [44,45,46]. Furthermore, it is suggested that elevated ROS levels in tumor cells facilitate greater proliferation, given that proliferation requires the presence of ROS [47,48]. These ideas can be interpreted in terms of developing an anti-cancer strategy along two lines: either further increase oxidant levels in the cancer cell, forcing the cell to experience high levels of oxidative stress and ultimately causing death from this stress, or use high doses of antioxidants to inhibit proliferation altogether. Moreover, given the great diversity of cancer tumors, consisting of heterogeneous cells and different tumor environments [49], such a task is difficult to accomplish even theoretically [50,51,52]. First, it is difficult to accomplish due the fact that the antioxidant activity for each antioxidant is highly dose-dependent (antioxidant window), resulting in a rather narrow optimum of antioxidant concentrations that maximizes its antioxidant potency. However, while an antioxidant deficiency can be overcome by increasing its dose or using other antioxidants, an excess of the dose above the optimum can convert it into a prooxidant, that is, an initiator of oxidative cascades, which are already quite developed in cancer cells. A good example of the latter is the conversion of vitamin C (ascorbate), used in anticancer therapy, from an antioxidant (due to the oxidation of the double bond in the molecule) to a prooxidant (due to the formation of dehydroascorbate, which initiates oxidative stress) [53,54]. The lack of clarity in the definition of an antioxidant was overshadowed by the fact that ascorbate killed cancer cells, which is the primary goal regardless of the mechanism interpretation. In Table 1, we provide examples of substances that may have anticancer properties by altering the redox status of the cancer cell, indicating different targets. It should be noted that many agents are aimed at preventing the reduced state of glutathione and, in general, at inhibiting reductase activity.

Undoubtedly, there are a number of redox-dependent targets in tumor cells [49,50], but special attention should be paid to the redox dependence of proliferative processes in general and cancer cells in particular and the system of xenobiotic release from the cell (reviewed in [5,6]). Taking into account the numerous published works on the effect of redox status on cell proliferation (e.g., see [66]), we limited ourselves to a brief presentation of individual facts, again focusing on the fact that in the practical plane of using redox agents for the therapy of malignant diseases, there are serious problems listed above.

In general, cell proliferation is determined by intracellular and extracellular factors. To illustrate the complexity of tumor cell proliferation (cancer progression), we will limit ourselves to a few examples, particularly those related to changes in the levels and flux of metabolites involved in cellular energetics, demonstrating the critical influence of the redox status inside and outside the tumor on its growth. The tumor microenvironment is highly heterogeneous in cellular composition, with macrophages and T cells, specifically those designed to combat tumor cells, making up a large proportion. The tumor redox environment plays a critical role in cancer progression. The lactate/pyruvate ratio, corresponding to the NAD/NADH ratio [67], makes an important contribution to the overall redox potential in cancer cells. Redox status, through the degree of reduction of various electron acceptors, including cellular energy components such as NAD and coenzyme Q, is extremely important for the initiation and course of cell proliferation. As has been shown, activation of the glycolytic flux is essential for active proliferation. Even in the presence of oxygen during active proliferation, tumor cells prefer cytosolic glucose metabolism, ultimately producing lactate, although it would theoretically be possible to utilize the intermediate product, pyruvate, and send it into the more energetically favorable tricarboxylic acid cycle. However, in cancer cells, mitochondrial metabolism with pronounced respiration or without it does occur and it is essential for tumor progression [68,69,70]. This is apparently necessary to get rid of the cell of excessive NADH produced by glycolytic metabolism and to regenerate coenzyme Q, which occurs in the Q cycle of the mitochondrial respiratory chain, ultimately creating a more oxidized redox state within the cell. However, the redox state outside the cell, that is, in the tumor environment, also contributes to tumor progression. Cancer cells release lactate accumulated in glycolysis, which is captured by T cells and inside them is reduced to pyruvate by lactate dehydrogenase, dramatically changing the internal redox status of T cells to a more reduced one. This in turn inhibits the activity of glyceraldehyde dehydrogenase and phosphoglycerate dehydrogenase, thereby sharply reducing the glycolytic flux and therefore the proliferation of T cells necessary to replenish those lost in the fight against tumor cells [71].

It should be noted once again that there is a great chunk of data on the effect of redox status on proliferation, and their full description is beyond the scope of this work, given the experimental studies and reviews mentioned above (e.g., see [66]). Of course, this imposes requirements for searching for redox agents that change metabolism in the direction needed for therapy. However, often those agents that are attributed with the properties of affecting redox status can provide their therapeutic potential through other mechanisms, not related to the regulation of oxidant levels in cancer cells.

## 5. Uncouplers of Oxidative Phosphorylation in Cancer Therapy: Potential Mechanisms

Given the proven therapeutic potential of a number of mitochondria-targeted substances, much attention is being paid to substances called “mild” uncouplers of oxidative phosphorylation. These substances, by definition, reduce the membrane potential of the mitochondrial inner membrane, but not so significantly as to halt ATP synthesis at the expense of the remaining membrane potential. This is achieved by a moderate (mild) leakage of protons through the initially impermeable inner mitochondrial membrane, coupled with activation of respiration. The use of such substances has been shown to be promising in the treatment of a wide range of pathologies, including those involving oxidative stress, but not limited to them [72,73,74,75,76]. A precise explanation for the beneficial mechanism has not been provided, but most researchers agree that their effect is related to a generalized reduction in cellular oxidant levels. The source for this decrease was found in the mitochondrial respiratory chain, the components of which, as a result of accelerated respiration, are in a more oxidized state, and the probability of donating electrons from them to an oxygen molecule with the formation of ROS decreases [77,78] in a sigmoid mode depending on concentration [79,80,81]. According to this relationship, even a small drop in membrane potential can provide a significant reduction in the level of ROS in mitochondria.

Available data suggests that uncoupling induced by mild uncouplers has demonstrated potential for application in cancer therapy [82]. This data, combined with existing, controversial data on the use of antioxidants, cast doubt on the underlying mechanism of action of mild uncouplers, which relies on effect on cellular redox status.

However, it should be noted that, in addition to the reduction in ROS generation during uncoupling, there are a number of other factors that must be taken into account when considering the effects of uncouplers on cells.

First, by diminishing control from oxidative phosphorylation, uncouplers activate mitochondrial respiration, thereby reducing cellular oxygen concentration, which can trigger hypoxic signaling [83]. However, for the sake of objectivity, we still note that this factor may or may not be relevant to explain the anti-cancer action of uncouplers, since it is aimed both at the *survival* of the system under hypoxic conditions and at its destruction. Nevertheless, the contribution of this factor must be taken into account.

Second, as a consequence of the disruption of mitochondrial energetics caused by mitochondrial uncouplers or inhibitors, fragmentation (fission) of the mitochondrial reticulum occurs, forming small mitochondrial structures [25,84,85,86]. We have mentioned above that mitochondrial fragmentation can lead to an imbalance in the redox potential within cellular compartments. Instead of a fairly uniform redox environment achieved through equipotentiality of the mitochondrial reticulum, the environmental fragmentation occurs with each mitochondrial fragment having its own membrane and, consequently, redox potential. This fragmentation of the cellular redox space can lead to an imbalance in the functioning of coupled enzymatic systems in a cell volume, which can be fatal for the cell.

**Figure 5:**
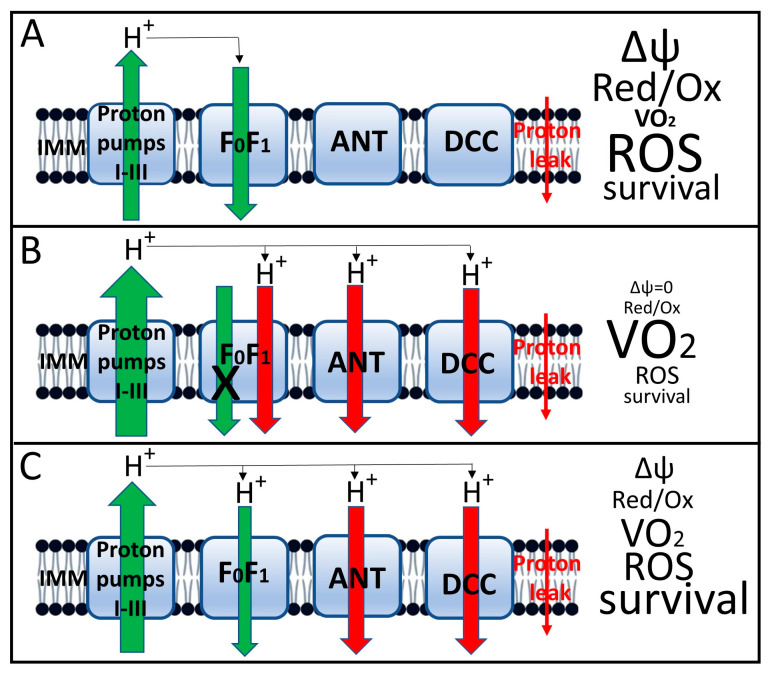
Induced uncoupling of oxidative phosphorylation and mitochondrial and cellular parameters associated with this process. (**A**), Tight coupling of oxidation (as expressed by proton flux through respiratory complexes I–III) and phosphorylation (backward proton flux through the ATP synthase complex, designated FoF1). The small red arrow on the right shows the nonspecific proton leak that drives respiration in state 4. This state is characterized by a relatively high membrane potential, a low respiration rate (designated VO_2_), moderate ROS generation and high host cell viability; (**B**), Complete loss of coupling induced by high concentrations of uncouplers. This state is characterized by loss of proton potential, failure of proton-transport-coupled with ATP synthesis in the FoF1 complex, maximal acceleration of respiration, low ROS production, and severe loss of host cell viability due to loss of oxidative energetics and decreased ATP levels. (**C**), moderate (mild) uncoupling, which maintains membrane potential at a level sufficient to ensure moderate ATP synthesis with decreased ROS production and increased cell viability. The size of the arrows indicates the relative level of proton flux, with green representing beneficial, energy-related fluxes and red representing proton leak not coupled to energy production. Uncoupler-induced leak occurs through the adenine nucleotide translocator (ANT) [89], dicarboxylate carrier [90] and FoF1 ATP synthase complex [76,88,91]. In the fully coupled state (A), leak through these proteins is either absent or minimal. It is possible that this leak is provided by uncoupling proteins (UCPs), which are not indicated in the diagram, since it only considers the leak acutely induced by uncouplers.

The third, and perhaps most important, result of uncoupling is a decrease in the transmembrane potential on the inner mitochondrial membrane [78]. (Figure 5 schematically shows the functioning of mitochondria under conditions of tight coupling and varying degrees of coupling with the characteristics accompanying each state). We emphasized above the critical importance of maintaining a constant, sufficiently high membrane potential as a prerequisite for normal cellular homeostasis [20,21,22]. As a result, a decrease in membrane potential is accompanied by a reversal of the activity of mitochondrial ATP synthase [87], which is aimed at maintaining membrane potential through ATP hydrolysis. This results in a futile proton cycle across the inner membrane, coupled with the consumption of cellular ATP until it is completely depleted, which is extremely dangerous for the cell and will cause its death by the mechanism of necrosis [88].

Signaling role of ATP in the cell, the concept of the “ATP meter” was put forth [92]. Membrane potential is a precursor to ATP synthesis, but its importance in the cell is probably not limited to this, since the insistent requirement for its existence cannot be explained by its involvement in energetics. In addition to its existence on the inner mitochondrial membrane, its presence has been noted on various membranes, where it regulates a multitude of processes (reviewed in [93]). If we turn to the precursors of mitochondria, bacteria, the membrane potential existing on the coupling membrane plays other non-energetic functions, including signaling. It can ensure the motility of bacterial cells by directly activating the motor that controls the movement of cilia, regulate the activity of transmembrane transport, participate in antibiotic resistance, determine pH homeostasis, and participate in cell division (in particular through its participation in the distribution of proteins and dynamic communication between individual cells [94,95], reviewed in [96]. The role of membrane potential in bacterial taxis deserves serious attention [97,98,99], which for the first time gave rise to the assumption of the involvement of membrane potential in signaling processes. This assumption was extended to the idea that the sensors of membrane potential in the cell are ion channels carrying a special domain called the voltage-dependent domain (VSD) (e.g., see [100]). Attempts have been made to search for VSD domains in mitochondrial ion channels [101], and indications have been obtained that the voltage-dependent anion channel (VDAC) of the mitochondrial outer membrane has a region with a lysine at position 12 (K12), which may control the channel’s voltage sensitivity [102]. These studies point to the possibility of membrane potential sensors in mitochondria, which could change the understanding of many cellular and mitochondrial processes, including a full explanation of the effects of uncouplers on eukaryotic cell physiology.

However, we would like to highlight the limitations of our discussion of the potential use of uncouplers of oxidative phosphorylation in oncology. The main challenge will be selecting uncouplers and their concentrations that will ensure *not cell survival* (as described for mild uncouplers), but *rather their killing*, which we can achieve based on the cellular changes (ATP depletion, mitochondrial fragmentation, and dramatic redox potential imbalance across the cell volume) we described. Mitochondrial-targeted uncouplers, particularly those that can be retained in tumor cells, may be promising [103,104,105].

## 6. Conclusions

The purpose of this analytical review is not to provide a detailed description of the relevance of redox potential to cellular function in health and disease. Given that oncological and cardiovascular diseases are the most significant contributors to mortality statistics, we only briefly touched on the importance of assessing and changing redox potential during the development of these pathologies, devoting more time to the role of redox potential changes in oncogenesis. However, the primary goal of our work was to gather information on avoiding oversimplification of this important science, which contains aspects that must be considered and discussed, even if no solutions exist to date. First and foremost, the main problem is the heterogeneity of redox potential within the cell and its membrane potential within tissue, while only average values of redox potential are usually assessed. Perhaps the optimism in this assessment lies in future technical improvements in redox potential recording, providing maximum spatial resolution across a biological sample while utilizing the maximum possible use of internal indicators.

## Figures and Tables

**Table 1:** Examples of chemotherapeutic compounds regulating glutathione and thioredoxin status in cancer cells.

Compounds	Target	Mechanism	Result	Refs
BSO (buthionine sulfoximine)	Glutathione synthetase (GS)	Inhibition of glutathione synthesis	Increased sensitivity to chemotherapy and radiation therapy	[55]
BCNU (carmustine) (1,3-bis(2-chloroethyl)-1-nitrosourea)	Glutathione reductase (GR)	Inhibition of glutathione reductase	In animals (glioma induction in rats/mice), BCNU significantly reduced tumor volume and increased survival, which became the basis for subsequent clinical trials in gliomas	[56,57,58]
VNP40101M (cloretazine) (1,2-bis[methylsulfonyl]-1-[2-chloroethyl]-2-[(methylamino)carbonyl] hydrazine)			Carbamoylates proteins, including O^6^-alkylguanine-DNA alkyltransferase (AGT/MGMT), which blocks repair and enhances cytotoxicity	[59]
2-AAPA (2-acetylamino-3-[4-(2-acetylamino-2-carboxyethylsulfanylthio carbonylamino)phenylthio carbamoylsulfanyl] propionic acid)			G2/M delay, redox homeostasis disturbance and antiproliferative effect	[55,60]
RSL3 (methyl (1S,3R)-2-(2-chloroacetyl)-1-(4-methoxycarbonylphenyl)-1,3,4,4a,9,9a-hexahydropyrido[3,4-b] indole-3-carboxylate)	Glutathione peroxidase (GP)	GPx4 inhibitor. Ferroptosis inducer in tumor cells	In colorectal cancer, RSL3 induces ROS, reduces GPX4 and causes cell death, and suppresses xenograft growth in mice	[61]
Erastin	Membrane cystine/glutamate transporter Xc^−^. Glutathione peroxidase	Ferroptosis inducer in tumor cells. Suppresses GP activity	Enhances the effect of radiation therapy	[62]
Auranofin gold(I) (phosphine complex)	Thioredoxin reductase	Inhibitor of thioredoxin reductase	Increases the sensitivity of tumor cells to radiation therapy	[63]
Hydroxyurea (HU) (hydroxycarbamide)	Ribonucleotide reductase	Inhibition of DNA synthesis	Increases the sensitivity of cells to chemotherapy, for example, by bleomycin	[64,65]

## Data Availability

No new data were created or analyzed in this study. Data sharing is not applicable to this article.
